# Processes of Fatigue Destruction in Nanopolymer-Hydrophobised Ceramic Bricks

**DOI:** 10.3390/ma10010044

**Published:** 2017-01-06

**Authors:** Stanisław Fic, Andrzej Szewczak, Danuta Barnat-Hunek, Grzegorz Łagód

**Affiliations:** 1 Faculty of Civil Engineering and Architecture, Lublin University of Technology, 40 Nadbystrzycka Str., Lublin 20-618, Poland; s.fic@pollub.pl (S.F.); a.szewczak@pollub.pl (A.S.); d.barnat-hunek@pollub.pl (D.B.-H.); 2 Faculty of Environmental Engineering, Lublin University of Technology, 40B Nadbystrzycka Str., Lublin 20-618, Poland

**Keywords:** low-cycle fatigue strength, surface hardness, absorbability, hydrophobisation, sonication

## Abstract

The article presents a proposal of a model of fatigue destruction of hydrophobised ceramic brick, i.e., a basic masonry material. The brick surface was hydrophobised with two inorganic polymers: a nanopolymer preparation based on dialkyl siloxanes (series 1–5) and an aqueous silicon solution (series 6–10). Nanosilica was added to the polymers to enhance the stability of the film formed on the brick surface. To achieve an appropriate blend of the polymer liquid phase and the nano silica solid phase, the mixture was disintegrated by sonication. The effect of the addition of nano silica and sonication on changes in the rheological parameters, i.e., viscosity and surface tension, was determined. Material fatigue was induced by cyclic immersion of the samples in water and drying at a temperature of 100 °C, which caused rapid and relatively dynamic movement of water. The moisture and temperature effect was determined by measurement of changes in surface hardness performed with the Vickers method and assessment of sample absorbability. The results provided an approximate picture of fatigue destruction of brick and hydrophobic coatings in relation to changes in their temporal stability. Additionally, SEM images of hydrophobic coatings in are shown.

## 1. Introduction

The process of destruction of porous building materials caused by changes in temperature and moisture is a major problem in modern materials science reported in numerous works [1,2,3]. These environmental factors exert strains and stresses in the material, reducing their durability and lowering the comfort of the use of constructed buildings [4,5]. This issue mainly pertains to porous materials, which are commonly used in civil engineering such as wall or façade elements [6,7], as well as insulation materials [8]. The reduction of material durability caused by cyclic external effects is referred to as fatigue strength [9,10,11].

In line with other studies [12,13], placement of an environmental load, e.g., temperature gradient or moisture, against a material causes plastic (permanent) strains, which determine the behaviour of fatigued material. The effect of these environmental factors is defined as low-cycle fatigue strength. At a low-cycle load, fatigue is a progressive development of material damage due to multiple, repetitive external and internal environmental impacts. The studies conducted by, Wardeh and Perrin [4] and Matsumoto et al. [14], mainly concern determination of the impact of low temperature on the deterioration of construction materials, whereas other studies [12,15,16] describe the effects of dampness on their internal and external structure. These phenomena are usually unfavourable, because they not only lower the thermal insulation properties, but also cause progressive degradation of material, and consequently, its destruction. Bricks, being a ceramic material, are characterised by an internal porous structure with a developed network of pores and capillaries, as well as damage in the form of micro-scratches and cracks created in the course of burning of clay and other materials [17,18,19]. The capacity of the capillary action of water in a brick and retaining moisture in its volume was the subject of numerous studies [20,21]. According to Tittarelli, when the temperature is lowered to 0 °C, the water contained in the pores increases its volume by approximately 9% and leads to formation of defects through relaxation of the internal structure of the materials [22]. The destruction process proceeds likewise at cyclic drying at relatively high temperatures and through filling of pores with water, which changes the strain and stress state, e.g., shrinkage and swelling. 

**Figure 1:**
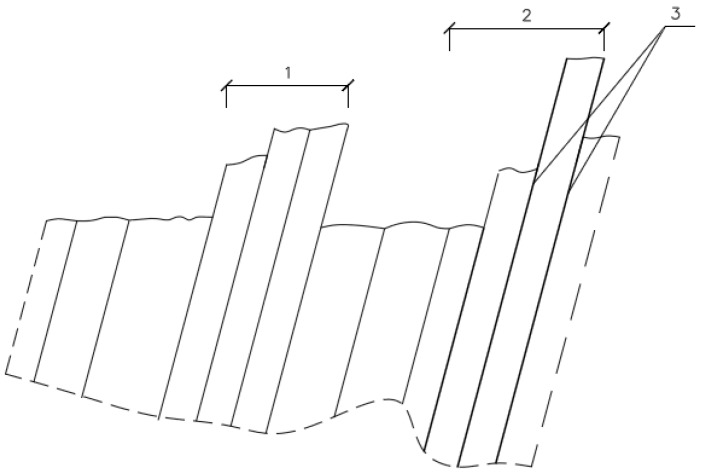
Scheme of the formation of slip bands and slip lines at a low-cycle load: 1 and 2 and slip bands; and 3 is a slip line [13].

Plastic strains manifest themselves as slip lines and slip bands, as shown in Figure 1. 

The slip line can be defined as a slip trace on the free surface in one slip plane. In turn, slip bands are made up of slip lines formed along the planes through dislocation.

**Figure 2:**
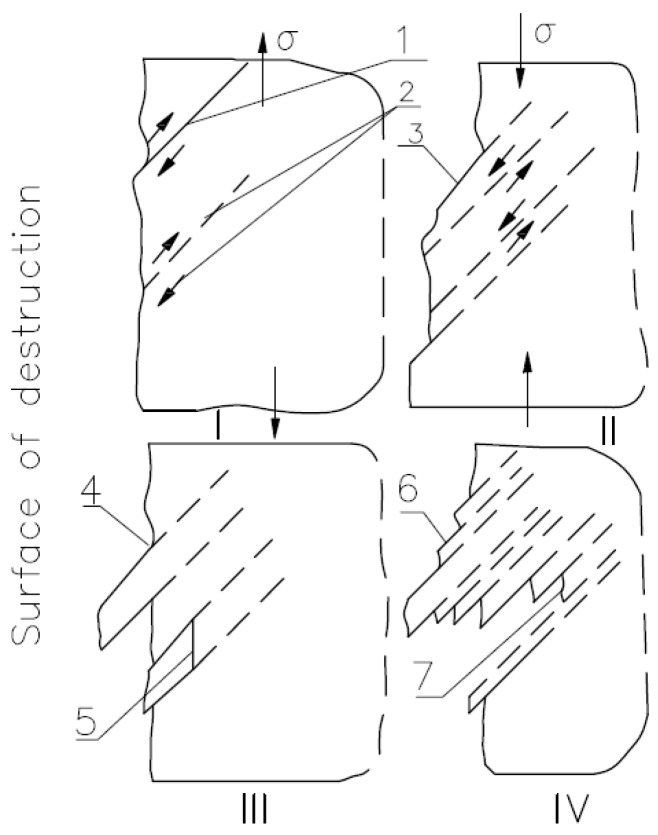
Scheme of formation of extrusions and intrusions: 1–3 are plane deformations; 4 and 5 are surface change; 6 is the formation of extrusions; and 7 is the formation of intrusions, II, III, IV—stages of intrusion and extrusion [23].

As specified by Fic et al., slip bands can be defined as extrusions and intrusions [23]. Extrusions are elevations above the surrounding of the material fragment and intrusions are depressions in the slip bands. The scheme of formation of extrusions and intrusions is shown in Figure 2.

According to several authors, micro-cracks are initiated and develop in slip bands as a result of concentrated strains (stress) and accumulation of energy in the planes between them [24,25,26]. The development of scratches is more clearly visible on the borders between the grains rather than inside the grains, since the dislocation occurs towards the boundaries of the grains, where faults and pores are formed at the interface of the solid phases, which is confirmed by the observations made by Hoła and Fic et al. [16,23]. Typically, microcracks are formed in the surface layer of an element [24,27], which is less durable than the internal structure of the material and, therefore, more susceptible to the local stress concentration caused mainly by technological defects of the material arising during the production process as well as stress (environmental) factors. While accounting for the impact of moisture and elevated temperature on the material, the Rehbinder effect described by Podgaetskii also needs to be taken into consideration [28,29]. This phenomenon involves a change (in the case of porous materials, a decrease) in the surface energy density in the solid phase (brick) as a result of adsorption of the active substance (water). The influence of water contributes to creation of additional micro-scratches and cracks on the surface of a brick.

In order to mitigate the occurrence of destructive processes in construction materials such as bricks, stone, or concrete, various impregnates and hydrophobisation are commonly employed solutions [30,31]. As reported other studies, the hydrophobising impregnates characterised by the highest efficiency mainly comprise polymer and nanopolymer compounds (depending on the size of particles) produced on the basis of silicones, silanes, and siloxanes [20,32,33]. Moreover, as reported by several authors, certain methods for modifying the composition and preparation of impregnates are employed in order to further improve their properties expressed by limiting either the water absorption or the free surface energy [21,30,34]. The particle size of organosilicon compounds affects the depth and the rate of penetration into the material structure. Silicone resins are characterised by the largest particles, which are circa 100 times larger than the particles of siloxanes [31].

In a nanopolymer-hydrophobised ceramic brick, the surface layer is reinforced by the formation of a surface film [35], in which case microcracks will both arise in the surface layer and reach a certain depth of the interior of the sample [24]. Further degradation of the material may occur when the top layer of the material is loosened and its inner layer is exposed to the further action of the loads. This necessitates protection of the surface of ceramic materials.

**Figure 3:**
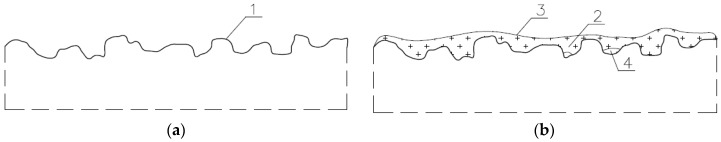
Scheme of the nature of mechanical adhesion: (**a**) brick surface before hydrophobisation; and (**b**) brick surface after hydrophobisation (1—rough surface of the brick; 2—glazed nanopolymer film; 3—rough surface of the glazed film; and 4—unfilled pores and air voids).

In the present study, we analysed samples of ceramic brick hydrophobised by immersion in a nanopolymer for 15 s, which resulted in formation of a thin film with a rough surface on the brick, as shown in Figure 3. The thickness of the film mainly depended on the surface roughness of the material.

According to numerous studies performed by, among others by Hazlett, Baldan, Kuczmaszewski, Rudawska, incorporation of a nanopolymer into the brick surface primarily occurs through mechanical adhesion, which is affected by the rheological properties of the impregnate, roughness and structure of the hydrophobised surface, and the thickness of the formed layer [36,37,38,39]. The depth of cavities has an influence on the strength of adhesion connection [40]. The Hagen–Poiseueille law and the capillary pressure equation describe the process of penetration of the polymer into material pores and capillaries, which was reported by Żenkiewicz [41]. According to Zare, adhesion is also affected by the shape memory, i.e., the capacity of the polymer layer to “remember” the shape of the base [42].

Other factors determining the structure of the film and its effectiveness in prevention of water penetration include chemical (in particular hydrogen) bonds and Van der Waals forces between the molecules of the polymer and the hydrophobised material, as described by Fic et al. and Van Oss et al. [21,43].

**Figure 4:**
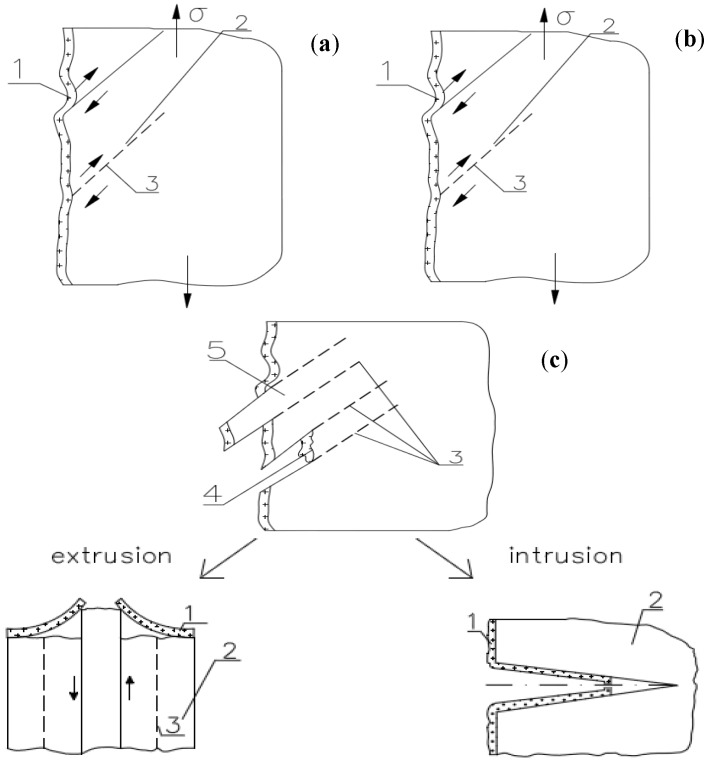
Scheme of destruction of the hydrophobising coating: (**a**,**b**) initial phase of destruction; and (**c**) intrusion and extrusion image (1—hydrophobising coating; 2—porous material; 3—slip plane; 4—film damage; 5—slip band; and σ—concentration of stress (deformations)).

**Figure 5:**
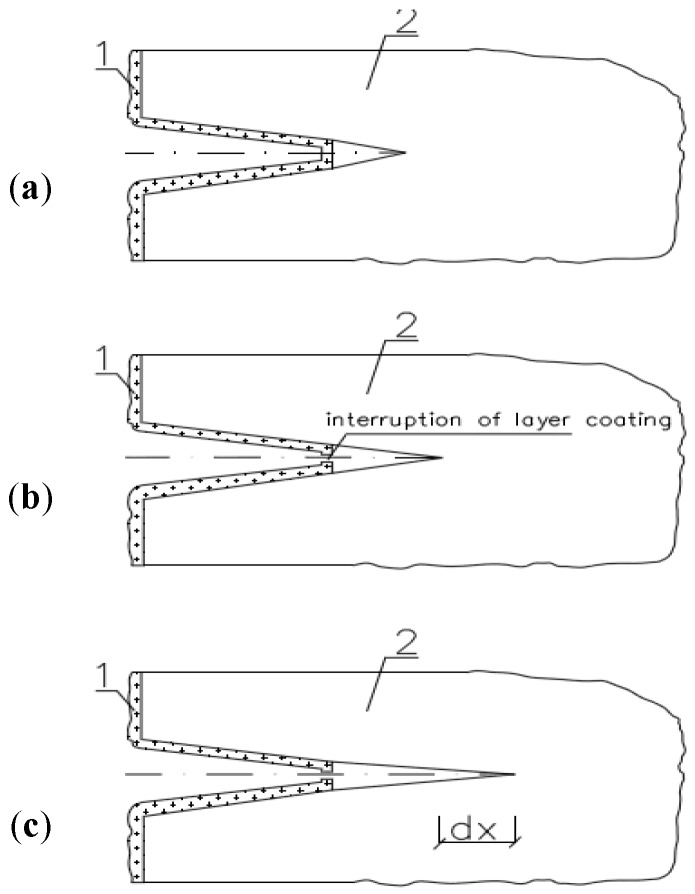
Scheme of propagation of a crack in the intrusion (1—hydrophobising coating; 2—material; and *dx*—propagation of the crack): (**a**) Initial phase of the process in hydrophobised material; (**b**) Phase of fatigue loading; and (**c**) Phase of the coating destruction and propagation of the crack (*dx*).

The glazed film-porous system is influenced by cyclic fatigue loads. Consequently, deformations arise on the surface; they initially cause damage to the surface layer of the film and induce formation of micro-cracks and scratches, thereby promoting water penetration into the material [44]. Secondary reorganisation of the film surface through the temperature and moisture effect is essential for increasing the effectiveness of protection. This process, however, is difficult to achieve, as it requires the use of specific additives to polymers, e.g., fillers. An important problem is the selection of the type of filler that would be compatible with the chemical composition of the polymer molecules. Degradation of the film in combination with formation of intrusions and extrusions on the brick surface ultimately causes the destruction of the material. The destruction model is shown in Figure 4 and Figure 5.

The article presents the destruction processes arising from low-cycle fatigue on the example of a ceramic brick hydrophobised with nanopolymers. The study carries on with the topic described in several papers [21,31,35], pertaining to the prospective modification of the composition of silicone-based hydrophobising agents with inorganic fillers. Low-cycle fatigue of the hydrophobic layer was induced by an experimental scheme involving an alternating impact of temperature and humidity on a ceramic brick hydrophobised with alkyl-siloxane nanopolymer. In contrast to the informations reported in studies [45,46], where the simulation of porous material fatigue was investigated by means of computer simulations, this study assumed the changes in water absorbability and surface hardness as an indicator of changes in the layer and brick properties. At present, no research has been conducted with the assumed methodology. The analysis of SEM photos was carried out in order to determine the changes on the brick surface (a similar SEM photo analysis can be found in study [47]).

## 2. Materials and Methods

The present research consisted of three stages of examinations: characteristics of ceramic bricks and formulations, method for sample preparation and test methods, and finally determination of the parameters of hydrophobised bricks.

### 2.1. Characteristics of Materials

The fatigue analyses were performed in 10 series, each comprising 6 samples excised from the ceramic brick.

Determination of the physical parameters was conducted according to PN-EN 1936:2010 and PN-EN 1389:2005 [48,49]. Density, apparent density, and total and open porosity were determined in the research. The following results were obtained: density *ρ* = 2.55 g/cm^3^, apparent density *ρ_a_* = 1.42 g/cm^3^, open porosity *P_o_* = 22.12%, and total porosity *P* = 31.51%.

The samples were dried to constant weight, weighed, and hydrophobised with preparations based on the following:

1. Oligomeric dialkyl siloxanes characterised by the following physical parameters:
  - viscosity η = 1.08 × 10^−3^ Pa·s;
  - surface tension σ = 23.51 × 10^−3^ N/m;
  - surface tension-to-viscosity ratio η/γ = 21.77;
  - density at 20 °C—*ρ* = 0.80 g/cm^3^.
2. Aqueous methyl silicone resin in potassium hydroxide characterised by:
  - preparation-water ratio 1:8;
  - viscosity η = 0.98 × 10^−3^ Pa·s;
  - surface tension γ = 77.24 × 10^−3^ N/m;
  - surface tension-to-viscosity ratio η/γ = 78.82;
  - density at 20 °C—*ρ* = 1.03 g/cm^3^ [31].
3. Parameters of water for comparison:
  - viscosity η = 0.89 × 10^−3^ Pa·s;
  - surface tension γ = 72 × 10^−3^ N/m;
  - surface tension-to-viscosity ratio η/γ = 80.90;
  - density at 20 °C—*ρ* = 0.99 g/cm^3^.

The excised brick samples were hydrophobised by full immersion in appropriately prepared polymer solutions based on small-molecule dialkyl siloxanes and/or silicones.

Series of samples used in the investigations and the mode of preparation thereof (with the formula of the hydrophobising preparation) are shown in Table 1.

### 2.2. Methods

The disintegration of the solution was carried out with the use of a stationary laboratory homogeniser with a 400-W ultrasonic processor UP400 (Hilescher, Tieltow, Germany). The device has been certified in accordance with DIN-EN ISO 9001.

All samples were stored for 14 days in laboratory conditions at a temperature of 20–22 °C and 50%–55% air humidity.

Sonication was applied as one of the effective methods for changing polymer viscosity and surface tension. It was also an initiator of sonochemical reactions between the molecules of the polymer and the filler [34]. Changes in the basic rheological properties of polymers induced by partial disintegration of the primary polymer chain structure and cavitation exerts and impact on the ability of polymers to penetrate and fill the pores of the ceramic material completely and, consequently, to form a tighter hydrophobising coating.

The fatigue of the ceramic bricks was achieved via a cyclic alternating process of drying of the samples at a temperature of 100 °C until maximum saturation. Surface hardness was examined after 10, 20, 25, 50, and 60 cycles. Simultaneously, the samples were weighed after each cycle to determine changes in their absorbability induced by the modelled low-cycle fatigue.

The investigations consisted in measurements of the hardness of the surface of the hydrophobised samples from all the series with the Vickers method using the Zwick–Roell device at a load range of 0–200 N (Zwick–Roell, Ulm, Germany). The performed measurements were automatically read based on the indentation of a right square-based pyramid applied to the material surface. Hardness was defined as the ratio of the force applied to the sample surface to the surface of the indentation. For each sample, 25 measurements of hardness were performed at a base load of 10 N.

Analysis of the distribution of the hydrophobic coating in the pores of the ceramic brick in SEM was carried out. The observations were compared with the images of the standard brick.

The method for preparation of the samples for scanning microscopy analyses excludes the possibility of formation of defects related to the sample surface and is considered non-destructive to the microstructure of the material or its coatings. Sometimes defects appearing on the sample surface are generated by the beam of energy applied to the sample. However, this is a clear optical effect, which can be observed on a computer screen. Therefore, low-energy systems generating a low vacuum and a low energy beam should be used. Such research methodology was adopted during the analyses of the ceramic bricks presented in the article

### 2.3. Determination of the Properties of Hydrophobised Bricks

**Figure 6:**
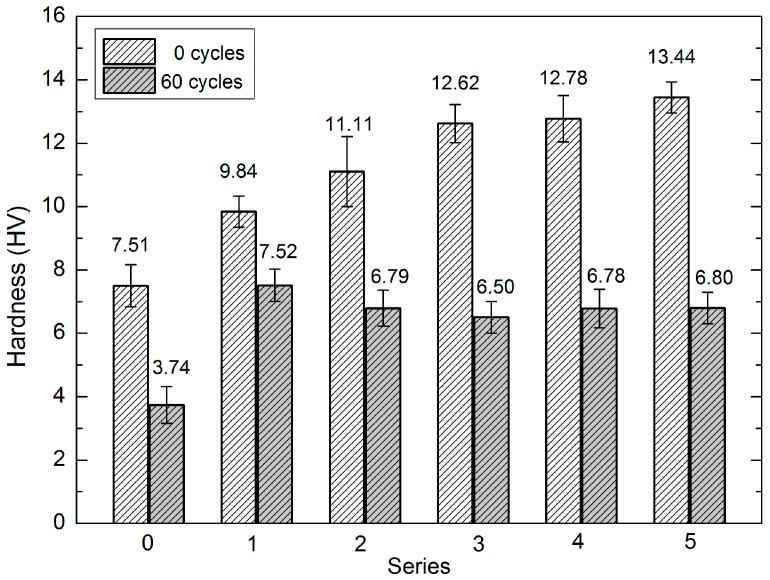
Hardness before and after 60 fatigue cycles in Series 0–5 brick samples.

The surface hardness of the analysed samples was assessed using a fragment of a non-hydrophobised ceramic brick as a standard. Measurement of the surface hardness of samples containing a sonicated and silica-supplemented nanopolymer solution revealed significant changes in the physico-mechanical properties of the analysed surfaces. The results of the measurements of the surface hardness (after statistical verification) are presented in Table 2 and Figure 6.

**Figure 7:**
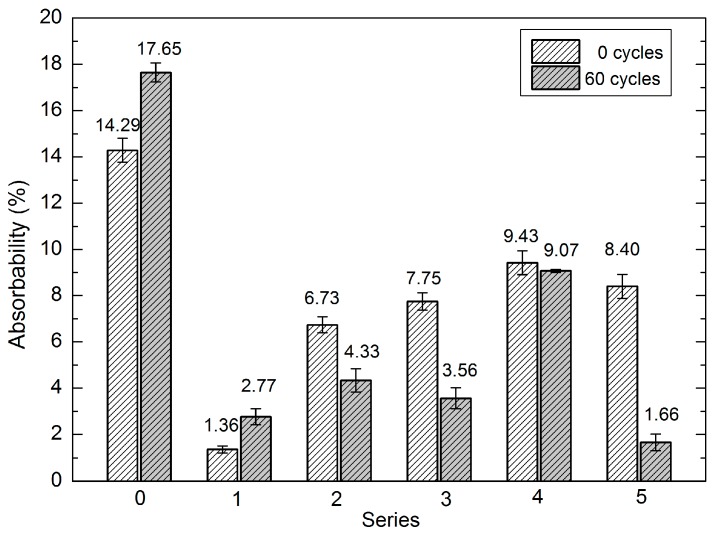
Absorbability before and after 60 fatigue cycles in Series 0–5 brick samples.

The results of the changes in the absorbability of the series induced by hydrophobisation and sonication are shown in Table 3 and Figure 7.

**Figure 8:**
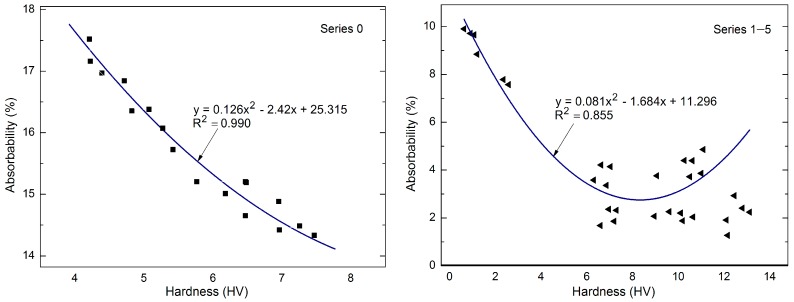
Correlations between the hardness and absorbability of standard bricks (Series 0) and bricks with a nanopolymer (Series 1–5).

There were correlations between the hardness and absorbability of the standard bricks (Series 0) and those with the modified nanopolymer (Series 1–5). A linear regression model with a single input variable was used and the results are presented in Figure 8.

**Figure 9:**
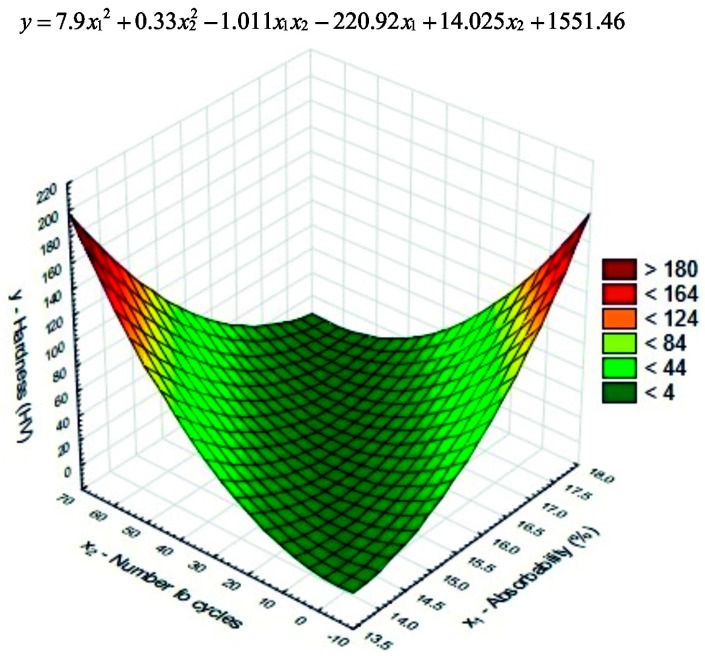
Correlation between hardness *x*_1_ (absorbability) and *x*_2_ (the number of fatigue cycles) in non-hydrophobised bricks (Series 0).

**Figure 10:**
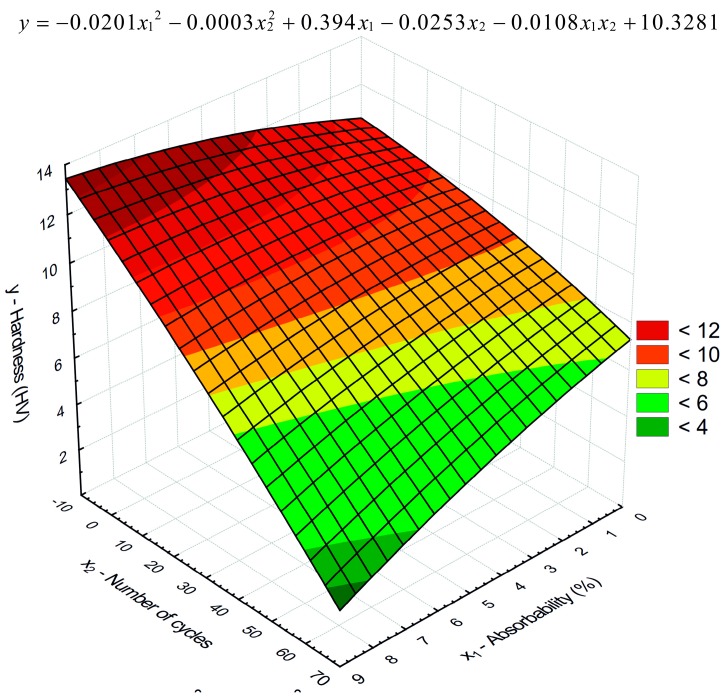
Correlation between hardness *x*_1_ (absorbability) and *x*_2_ (the number of fatigue cycles) in hydrophobised bricks with addition of 0.5%; 1.0% and 1.5% nanosilica and sonication (Series 3–5).

A more complex experimental model with two variables is proposed below (Figure 9 and Figure 10). The second-degree model functions were determined using the least squares method in Statistica software [50]. The complex models were designed to reveal the effect of hydrophobisation on the physical properties of the ceramic material.

**Figure 11:**
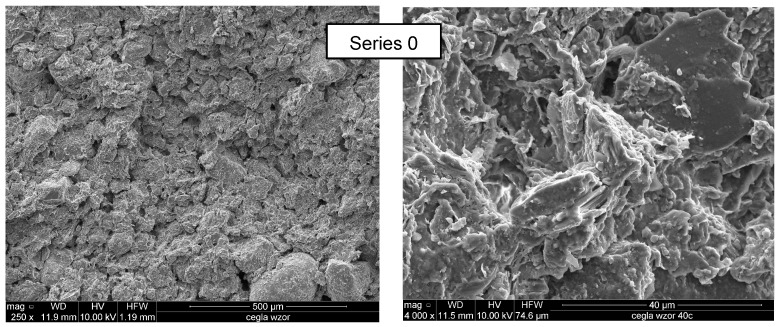
Structure of non-hydrophobised brick (250 and 4000×).

**Figure 12:**
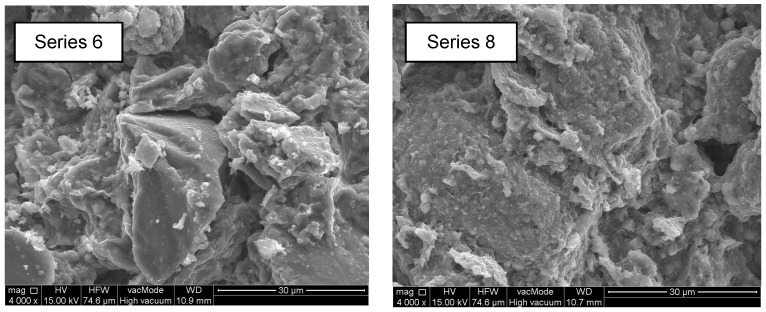
Structure of brick hydrophobised with methyl silicone resin (Series 6) and methyl silicone resin with addition of 0.5% microsilica (Series 8) (4000×).

**Figure 13:**
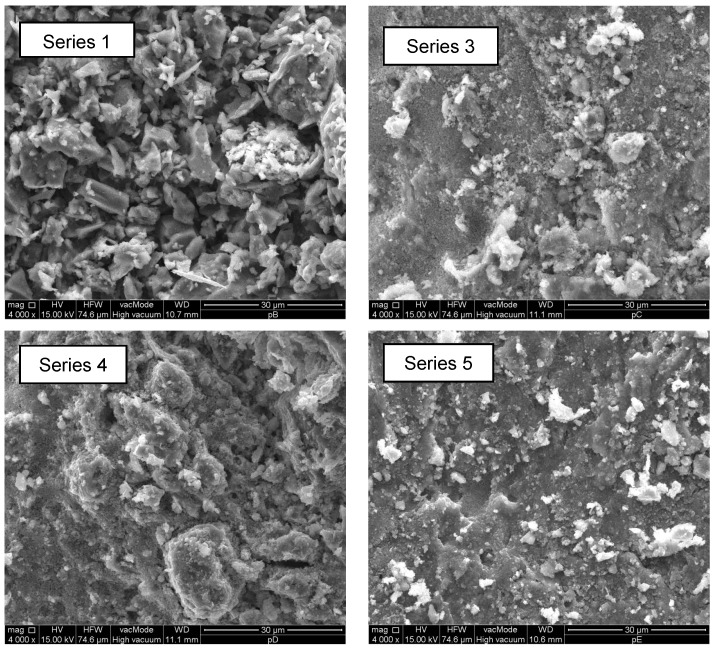
Structure of brick with polymers based on dialkyl siloxanes (Series 1) and with 0.5%, 1%, and 1.5% nanosilica addition (4000×).

Microscopic images showing the structure of the standard brick before hydrophobisation are shown in Figure 11. The texture of the silicone resin on a ceramic brick fracture is presented in Figure 12 and Figure 13.

## 3. Discussion

The investigation results indicate that the adopted model of fatigue tests in the hydrophobising coatings of ceramic materials can be successfully used for determination of the effectiveness of the coatings. Cyclic application of high temperature (in the range of 0–100 °C) and saturation of brick pores with water efficiently models the work of these elements exposed to prevailing weather conditions. The two parameters, i.e., surface hardness and absorbability, adopted for comparison as the basic factors characterising physico-mechanical changes in the analysed materials, are appropriate indicators of the effectiveness of the hydrophobising preparation applied, which are easy to examine and assess. The fatigue tests have demonstrated a positive influence of the addition of silica nanoparticles on these parameters in terms of the capacity of coating reorganisation and reconstruction the during the fatigue cycles. Obviously, ceramic materials are destroyed over time by temperature and humidity and the process is largely related to their porous structure. However, a certain degree of resistance of these materials to deformations leading to formation of extrusions and intrusions and, consequently, scratches and cracks resulting in degradation of the material can be achieved. In the sonochemical reaction initiated by ultrasonic energy, large silicone molecules are not easily incorporated into nanosilica molecules, which do not penetrate deep into the silicone internal structure but remain in its upper layer. This is reflected in reinforcement of the layer and higher susceptibility to damage caused by formation of intrusions and extrusions, which is associated with its more fragile structure. The incorporation of nanosilica into silicone chains is also influenced by attachment of alkyl groups to silicon atoms, which impair the formation of hydrogen bonds.

Nanosilica is incorporated as an element of the siloxane chain, and penetration of the new polymer into the pores of ceramic bricks occurs as well as cross-linking of the hydrophobising coating in the material structure. Consequently, the film is more flexible, which is reflected in reduction of its mechanical strength due to the plasticity of the deformations.

### 3.1. Hardness of Hydrophobised Bricks

The investigation results indicate that surface hardness and changes therein are closely associated with the type and method for preparation of the hydrophobising preparation. In samples hydrophobised with the dialkyl siloxane preparation, the hardness value declined along the number of the fatigue cycles. In turn, samples hydrophobised with the methyl silicone resin exhibited an initial increase in the hardness (with the exception of the Series 10 samples) and a decline in its value after 20 cycles. The highest initial hardness value (13.33 HV) was noted in the Series 5 samples; in comparison, the highest surface hardness value in samples hydrophobised with the aqueous silicone solution was noted in Series 8 (11.73 HV). The rate of the changes occurring in the hardness, i.e., the mechanical strength of the samples, was varied. There was a steady 50% decrease in the hardness of the ceramic bricks after 60 cycles; however, this relationship was not obvious in the case of the hydrophobised samples (Table 1 and Figure 6). In each series, the interval between cycle 20 and 25 turned out to be crucial. A higher rate of the surface hardness decline was noted during this period in Series 1–5. In turn, the hardness value in Series 6–9 increased in this period in comparison with the initial value (maximum by 23%). Only the Series 10 samples exhibited the same change as samples 1–5. An interesting phenomenon was observed for Series 9, for which the final hardness reached the maximum value (9.60 HV) and was only slightly lower than the initial value. In contrast, this value decreased by 49% in Series 5. Nevertheless, the mean final value was always by 27%–60% higher than the final hardness of the non-hydrophobised brick after 60 cycles, which suggests that the stability of the hydrophobic coating was still relatively high (Figure 6).

### 3.2. Absorbability of Hydrophobised Bricks

Neither the siloxane coating nor the silicone coating lose their properties from the beginning of fatigue tests. In turn, one can observe their capability of cyclic reinforcement and sealing of the structure, damage to the coating delaying degradation, and cross-linking of polymer chains on the brick surface, which is enhanced up to a certain moment. In extreme cases in Series 5, the ability of the coating to protect the material against water penetration was stabilised. Consequently, sample absorbability after 60 cycles was only 1.88%; furthermore, the samples exhibited relatively high surface hardness, compared with the final hardness of the non-hydrophobised brick. The mean absorbability of the non-hydrophobised bricks was ca. 14% at the beginning of the investigations. It gradually increased during the consecutive cycles and reached 17.76% after 60 cycles (Table 3, Figure 7 and Figure 9). The lowest initial absorbability was found for the Series 1 samples. Depending on the series of the hydrophobised samples, the changes in absorbability had varied nature and rates. The absorbability declined until the period between cycle 20 and 25 in Series 1, 2a, and 3–10 and even up to cycle 25 in the case of Series 1a and 2. After this period, absorbability increased in each series at different rates. The absorbability of the bricks hydrophobised with the dialkyl siloxane preparation slightly increased (maximum by 3.57%), whereas the maximum increase exhibited a higher rate in the case of the silicone preparation and was estimated at 5.8%. In turn, the lowest absorbability value after 60 cycles was noted for the Series 5 samples (Figure 7).

Materials with no hydrophobic coating and those hydrophobised with the non-modified polymer were characterised by a steady absorbability increase at prolonged impregnation, which was confirmed in other studies reported by Barnat-Hunek [31,32]. There was a clear effect of the silica addition on the absorbability decline in the initial fatigue cycles. A combination of different additives of the same type, such as organosilicon compounds in this case, can have a beneficial effect on water resistance, which was confirmed by Van Gemert and co-authors [44].

### 3.3. Correlation between Absorbability and Hardness of Hydrophobised Bricks

There were correlations between the hardness of the polymer coating and the brick absorbability before, during, and after the fatigue cycles in the case of the bricks hydrophobised with the nanopolymer (Series 1–5) (Figure 8). Correlation presented there is characterised by good coefficient of determination, *R*^2^ = 0.855.

The simple experimental models with one input variable have confirmed that surface hardness can be a measure of brick absorbability after hydrophobisation without and with application of silica modification. No similar correlations were found for Series 8–10 hydrophobised with soluble silicon. Modification of the polymer with nanosilica by sonication resulted in an absorbability decline in the initial 10 or 20 fatigue cycles followed by damage to the polymer coating caused by material fatigue, which led to increased absorbability, while the material hardness decreased linearly from the beginning of the investigations (Table 2 and Table 3). This demonstrates a highly positive effect of the polymer modification with nanosilica and brick stability until ca. 20–25 cycles; afterwards, the polymer coating is damaged.

Next, we presented models (Figure 9 and Figure 10) showing the correlations between such ceramic material traits as absorbability and polymer coating hardness and the number of fatigue cycles. The second-degree equations presented in Figure 9 and Figure 10 indirectly define corrosion resistance of the ceramic material before (Series 0) and after hydrophobisation with the silica-modified polymer (Series 3–5).

### 3.4. Microscopic Observations of Hydrophobised Bricks

The structure of the standard brick exhibited irregularities, fissures, and numerous pores with varied diameters (Figure 11). This indicates high absorbability and vapour permeability.

Macromolecular siliconates (Figure 12 Series 6) produced an evenly distributed coating, which thoroughly covered the structure of the bricks. The methyl silicone-resin coating was composed of tiny plates, which did not seal the brick pores and thus did not impede diffusion of vapours and gases.

After sonication and mixing with 0.5% silica, the water-dilutable preparation formed a tighter coating, which completely covered the entire brick structure (Figure 12 Series 8). The SEM image at 4000× multiplication shows tiny spheres forming a uniform coating. It was 18% harder than the coating without the nanosilica addition and sonication in Series 6, which was confirmed by the analysis of hardness presented in Table 2. However, it did not ensure increased water resistance. The absorbability of the nanosilica-containing coating was 22% higher after 14 days of the analysis than the absorbability of the Series 6 bricks. However, a positive impact of the nanosilica-based modification of the polymer was observed after 60 fatigue cycles at alternating impregnation and drying. In this case, the absorbability of the bricks with 0.5% nanosilica was 21.7% lower than that of bricks with methyl silicone resin.

All polymers based on small-molecule dialkyl siloxanes with 0.5%, 1%, and 1.5% nanosilica addition coated on the brick samples produced a continuous and evenly distributed film with no discernible scratches or cracks. The non-modified preparation in Series 1 formed a vapour permeable silicon coating composed of tiny, sharp polysiloxane gel plates. These properties of the silicone resin should ensure good water resistance and hydrophobisation efficiency, which was confirmed by the analysis of absorbability, which exhibited a very low value of 0.84%. Similar observations were reported in the article by Barnat-Hunek et al. [31].

The coating containing dialkyl siloxanes with nanosilica is characterised by a fine-pore microstructure and is composed of very small spheres forming an excessively dense gel, which fully covers the mineral components and pores in the brick. The distribution of nanosilica-modified preparations reduces their effectiveness in comparison with the standard preparation (Series 1). The absorbability of the Series 1 brick was by 89%–91% lower than that of brick with the modified coating (Series 3–5). However, as in the case of the methyl silicone resin in Series 8, the absorbability value in Series 5 after 60 fatigue cycles was by 60.2% lower than that in Series 1. No such absorbability decrease was noted in the Series with 0.5% and 1% nanosilica. The excessive density of the polysiloxane gel formed in the structure of the material prevents its precise adhesion to all minerals contained in the brick. It can be assumed that voids that are not fully covered by the thin layer of the hydrophobic coating are formed. This is reflected in the high absorbability, compared with that of the non-modified hydrophobic coating (Series 1). In turn, compared with the standard non-hydrophobised brick, the absorbability of these bricks is by 40% lower.

The ineffectiveness of the dense, tight hydrophobic coating was reported in other articles [30,32]. The effectiveness of hydrophobisation can be ensured by thin polysiloxane gel coatings, which accurately cover all mineral components material without sealing the pores required for vapour permeability. Such coatings are more resistant to damage and do not cause formation of voids, in which water, water-soluble salts, and swelling ice can accumulate, thereby leading to flaking and destruction of the material.

## 4. Conclusions

Based on the analysis of the research results, the following conclusions are proposed:

(a) Increased mechanical and fatigue strength, e.g., resistance to damage induced by the tests, was achieved by the coating based on the aqueous silicon solution, which is associated with the chemical traits of silicones and the physical properties of the film.
(b) The sonochemical reaction proceeds more rapidly and is more effective in the case of incorporation of silica molecules into the internal structure of dialkyl siloxane-based nanopolymers. This is closely related to the size of siloxane molecules and the type of substituents in the main chain (besides alkyl groups, there are hydrogen atoms, which facilitates formation of hydrogen bonds).
(c) To compare conclusions (a) and (b), the fragility of the silicone coating makes it more susceptible to cracking, especially under the effect of the temperature gradient. The siloxane coating, which is more susceptible to deformation, simultaneously exhibits greater flexibility and higher susceptibility to roughness changes and formation of intrusions and extrusions; hence, it does not crack. Therefore, depending on the preparation applied, the image and course of the coating destruction vary.
(d) A very interesting phenomenon of secondary reorganisation of the hydrophobising coating structure caused by water movement in brick pores and capillaries caused by temperature changes was noticed. This phenomenon is highly advantageous from the point of view of the effectiveness of the hydrophobising agent formula.
(e) There is no close relationship between the amount of the silica filler added and the efficiency of hydrophobisation in terms of changes in the surface hardness and absorbability of the tested samples.
(f) Siliconates and small-molecule dialkyl siloxanes, which do not seal the structure and do not impair vapour and gas diffusion, form a relatively well-distributed coating in the brick structure. Dialkyl siloxane-based coatings modified with nanosilica form an excessive-density layer, which negatively affects brick absorbability. Modified methyl silicone-resin coatings exhibit an appropriate structure of polysiloxane gel, which was confirmed by its high effectiveness observed in the study.

## Figures and Tables

**Table 1:** Characteristics of Series adopted in the analyses.

	Sample Preparation Procedure		
Series	Addition of Nanosilica Relative to the Polymer Weight (%)	Polymer Disintegration by 15-min Sonication	Immersion of the Samples in the Preparation for 15 s *N-*Nanopolymer *S*-Silicon Solution
Series 0	-	-	-
Series 1	-	-	*N*
Series 1a	-	-	*N*—2x immersion
Series 2	-	×	*N*
Series 2a	-	-	*N*—2x immersion
Series 3	0.5	×	*N*
Series 4	1.0	×	*N*
Series 5	1.5	×	*N*
Series 6	-	-	*S*
Series 7	-	×	*S*
Series 8	0.5	×	*S*
Series 9	1.0	×	*S*
Series 10	1.5	×	*S*

×—sonication was used; - not applied.

**Table 2:** Surface hardness of hydrophobised bricks.

Series	Hardness (HV)					
	Number of Cycles					
	0	10	20	25	50	60
Series 0	7.77	6.73	6.70	6.02	4.27	3.92
Series 1	9.90	9.71	9.65	8.84	7.78	7.57
Series 1a	10.82	10.71	10.31	9.16	7.50	7.17
Series 2	11.30	11.21	10.47	9.27	7.24	6.83
Series 2a	13.03	12.95	10.86	9.60	6.10	5.40
Series 3	12.66	12.37	10.85	9.81	7.06	6.51
Series 4	12.88	12.53	10.32	10.24	7.49	6.94
Series 5	13.33	13.01	12.31	10.40	7.40	6.80
Series 6	9.62	9.73	10.63	9.50	9.38	9.35
Series 7	10.37	10.82	11.62	9.78	9.28	9.18
Series 8	11.73	11.94	12.37	9.79	7.79	7.39
Series 9	9.70	9.43	11.90	9.90	9.65	9.60
Series 10	10.05	10.01	9.55	8.85	7.60	7.35

**Table 3:** Impact of fatigue cycles on brick absorbability.

Series	Absorbability (%)					
	Number of Cycles					
	0	10	20	25	50	60
Series 0	14.12	14.53	14.93	15.32	17.27	17.76
Series 1	0.84	1.12	1.28	1.41	2.56	2.79
Series 1a	4.39	3.72	2.20	2.07	2.32	2.36
Series 2	6.86	5.86	4.40	3.76	4.14	4.21
Series 2a	4.62	1.33	1.20	1.32	1.92	2.04
Series 3	7.93	1.27	2.04	2.26	3.36	3.58
Series 4	9.29	5.74	5.55	6.06	8.61	9.12
Series 5	8.24	2.41	1.91	1.88	1.86	1.68
Series 6	7.21	4.19	3.87	4.13	5.43	5.69
Series 7	9.47	2.04	2.24	4.78	7.48	8.02
Series 8	9.25	2.17	1.37	1.81	4.01	4.45
Series 9	9.12	2.24	1.67	2.16	4.61	5.1
Series 10	8.95	2.58	1.95	2.62	5.97	6.64
